# Leadership Development Programs for Physicians: A Systematic Review

**DOI:** 10.1007/s11606-014-3141-1

**Published:** 2014-12-20

**Authors:** Jan C. Frich, Amanda L. Brewster, Emily J. Cherlin, Elizabeth H. Bradley

**Affiliations:** 1 Global Health Leadership Institute, Yale School of Public Health, New Haven, CT USA; 2 Department of Health Management and Health Economics, Faculty of Medicine, University of Oslo, PO Box 1089, 0318 Oslo, Norway

**Keywords:** physicians, leadership, program development, program evaluation, systematic review

## Abstract

**Background:**

Physician leadership development programs typically aim to strengthen physicians’ leadership competencies and improve organizational performance. We conducted a systematic review of medical literature on physician leadership development programs in order to characterize the setting, educational content, teaching methods, and learning outcomes achieved.

**Methods:**

Articles were identified through a search in Ovid MEDLINE from 1950 through November 2013. We included articles that described programs designed to expose physicians to leadership concepts, outlined teaching methods, and reported evaluation outcomes. A thematic analysis was conducted using a structured data entry form with categories for setting/target group, educational content, format, type of evaluation and outcomes.

**Results:**

We identified 45 studies that met eligibility criteria, of which 35 reported on programs exclusively targeting physicians. The majority of programs focused on skills training and technical and conceptual knowledge, while fewer programs focused on personal growth and awareness. Half of the studies used pre/post intervention designs, and four studies used a comparison group. Positive outcomes were reported in all studies, although the majority of studies relied on learner satisfaction scores and self-assessed knowledge or behavioral change. Only six studies documented favorable organizational outcomes, such as improvement in quality indicators for disease management. The leadership programs examined in these studies were characterized by the use of multiple learning methods, including lectures, seminars, group work, and action learning projects in multidisciplinary teams.

**Discussion:**

Physician leadership development programs are associated with increased self-assessed knowledge and expertise; however, few studies have examined outcomes at a system level. Our synthesis of the literature suggests important gaps, including a lack of programs that integrate non-physician and physician professionals, limited use of more interactive learning and feedback to develop greater self-awareness, and an overly narrow focus on individual-level rather than system-level outcomes.

## INTRODUCTION

High-quality health care increasingly relies on teams, collaboration, and interdisciplinary work, and physician leadership is essential for optimizing health system performance.^1^^–^ 3 The Accreditation Council for Graduate Medical Education (ACGME) has established common program requirements that include skills in interpersonal communication, quality improvement, and system-based practice.^4^ The CanMEDS Physician Competency Framework identifies and describes seven roles for physicians: medical expert, communicator, collaborator, manager, health advocate, scholar, and professional.^5^ As practice management, performance improvement, and system-based practice have become integral to residency training in the U.S.^6^^–^ 8, experts are calling for leadership development to strengthen practicing physicians’ leadership skills and competencies.^9^^–^ 15 The lack of a common conceptual framework, however, presents a challenge to the field. While leadership may be understood as motivating and influencing others to bring about change, management involves achieving specific results through planning, organizing, and solving problems.^16^ Some see leadership and management as separate systems of action, but in practice, the terms are often used interchangeably.^17^^,^ 18 Some leadership models focus on competencies required to fill leadership roles in a given organizational setting, such as self-awareness, technical and conceptual knowledge, and skills needed in leadership roles.^19^^,^ 20

Although the literature draws a distinction between leader development (building individual competencies) and leadership development (building collective capacity)^21^, the term "leadership development" often encompasses efforts to develop individual leaders as well as to build capacity for leadership within an organization.^22^^,^ 23 Leadership development can promote several key functions in organizations, such as performance improvement, succession planning, and organizational change, and the literature on leadership provides evidence that leadership development helps organizations to achieve their goals.^24^^,^ 25 Developing leadership capacity in groups and organizations includes promoting a culture of accountability and alignment.^22^^,^ 26 Target groups for leadership development may include individuals with or without formal leadership roles.^27^ Leadership development programs may be delivered internally, externally, or a combination of both, and recent surveys suggest wide variation in approaches to leadership development among health care organizations.^14^^,^ 15

We lack a synthesis in the scientific literature that summarizes recurrent themes and empirical evidence regarding physician leadership development programs. Accordingly, we sought to systematically review published medical literature on physician leadership development in order to characterize the settings, educational content, teaching methods, and learning outcomes achieved. Findings from this study may be useful for designing and evaluating future leadership development programs.

## METHODS

### Literature Search

**Figure 1:**
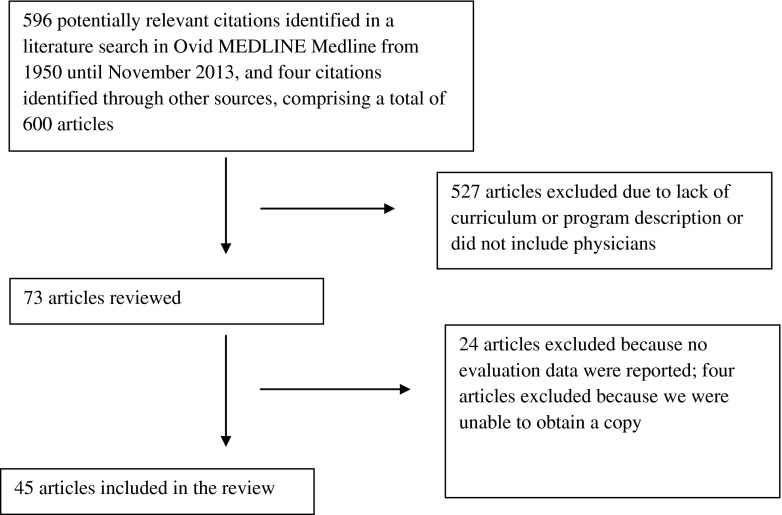
Identification and Selection Process for Articles Describing Leadership Development Course/Programs for Physicians.

We searched for relevant English-language studies published from 1950 through November 2013 using the Ovid MEDLINE electronic database. We initially identified articles using text keyword searches (e.g. “leadership development”or “physicians”). We then developed a comprehensive search strategy using Medical Subject Headings terms: (Physicians OR Physician executives OR Internship and Residency OR Medical staff) AND (Leadership OR Practice Management) AND (Program evaluation OR Program development OR Curriculum). The search identified 596 unique articles, and four additional articles were identified through other sources, comprising a total of 600 articles (Fig. 1).

### Eligibility Criteria

We included any peer-reviewed article that: (a) reported on an educational course, curriculum, or program designed to train physicians in leadership skills or expose physicians to leadership concepts, (b) outlined teaching methods used to achieve this goal, or (c) reported results from the evaluation of the course, curriculum, or program.

### Article Review Process

Two members of the research team (J.F. and E.C.) independently reviewed all titles as well as available abstracts. Of the 600 articles, we excluded 527 that were not relevant, such as articles that focused exclusively on medical students or nurses or articles that described programs intended only to build competencies in quality improvement or accounting skills. The full text of the article was consulted as needed. We identified 73 articles that described leadership development courses or programs, and we were successful in retrieving the full text for a total of 71 articles. Two researchers (J.F. and A.B...) reviewed these articles to determine their eligibility; 26 articles did not report evaluation findings and were therefore excluded, resulting in a final sample of 45 studies.

The article screening process was followed by independent abstraction of data by J.F. and A.B. from all 45 articles, using a structured data entry form with categories for setting/target group, teaching/learning method used, educational content, evaluation design, method, and outcome. Differences in categorization at the article screening and data abstraction stages were resolved through negotiated consensus.

### Data Analysis

We extracted curricular descriptors using the data entry form and recorded whether a curriculum was a one-time activity or an extended program, and to what extent didactic lectures/seminars, project work, group work, simulation, and multi-source/360-degree feedback tools were used. Leadership development may focus on personal growth, conceptual, or technical knowledge or skills^19^^,^ 20, and we recorded which of these aspects were covered by a program. Leadership development curricula may be evaluated using various outcome measures, including outcomes for individuals, groups or teams, organizations, networks, and societies.^28^ We used Kirkpatrick’s four-level evaluation model as a starting point for program classification.^29^ This model describes four evaluation levels: reaction (Level 1), knowledge (Level 2), behavioral change (Level 3), and system results (Level 4). In accordance with previous reviews on leadership development in the general leadership literature^24^^,^ 25 we differentiated between subjective and objective assessment of outcomes. Thus, seven categories were used to classify evaluation outcomes: reaction (Level 1), knowledge (subjective) (Level 2A), knowledge (objective) (Level 2B), behavior/expertise (subjective) (Level 3A), behavior/expertise (objective) (Level 3B), system results/performance (subjective) (Level 4A), and system results/performance (objective) (Level 4B) (Table 1).

**Table 1:** Typology of Evaluation Outcomes for Leadership Development Courses/Programs

Level	Label	Description
Level 1	Reaction	How participants feel about the program and their satisfaction with different components
Level 2A	Knowledge (subjective)	Principles, facts, attitudes, and skills learned during or by the end of the program, as communicated in statements, opinion, belief, or judgment by the participant or trainer
Level 2B	Knowledge (objective)	Principles, facts, attitudes, and skills learned during or by the end of the program, measured by objective means
Level 3A	Behavior/expertise (subjective)	Changes in on-the-job behavior perceived by participants, or global perceptions by peers or a superior
Level 3B	Behavior/expertise (objective)	Tangible results that evaluate changes in on-the-job behavior or supervisor rating of observable behaviors
Level 4A	System results/performance (subjective)	Organizational results perceived by respondents and group effectiveness perceived by subordinates
Level 4B	System results/performance (objective)	Tangible organizational results such as reduced costs, improved quality, and promotions

The typology is modified after Collins & Holton^25^ and Kirkpatrick.^29^

## RESULTS

### Setting and Target Group

Of the 45 studies that met the eligibility criteria,^30^^–^ 74 the majority (*n* = 34) reported on single residency/fellowship programs or programs for physicians, surgeons, or medical faculty. A minority (*n* = 11) of programs were multidisciplinary (Table 2). Authors cited the need to foster a nonthreatening participatory and exploratory environment as the primary reason for including physicians only.^58^^,^ 71 The desire for interdisciplinary learning, communication, and collaboration were cited as reasons for choosing a multidisciplinary approach.^40^^,^ 50 ^,^ 56 Among the 45 articles, 39 reported on courses and programs in the U.S. and Canada (Table 3).

**Table 2:** Features of 45 Studies of Leadership Development for Physicians

Feature		No. (%)
Educational setting		
	Single residency/fellowship program	19 (42)
	Physicians/surgeons/medical faculty	8 (18)
	Multiple residency/chief residency/fellowship programs	7 (16)
	Multidisciplinary programs	11 (24)
Educational aims		
	Skills	29 (64)
	Technical and conceptual knowledge	27 (60)
	Personal growth and self-awareness	9 (20)
Educational content		
	Leadership	35 (78)
	Teamwork	26 (58)
	Financial management	16 (36)
	Self-management	15 (33)
	Conflict management	13 (29)
	Quality improvement	12 (27)
	Communication	12 (27)
	Health policy/strategy	7 (16)
Teaching/learning methods*		
	Didactic lectures/interactive plenary seminars	36 (84)
	Group work	32 (74)
	Project work/action-based learning	17 (40)
	Simulation exercises	12 (27)
	Multi-source/360-degree feedback tool	3 (6)
Evaluation design		
	Pre/post	23 (51)
	Post	22 (49)
	Comparison group	4 (9)
	Quantitative only	32 (71)
	Qualitative only	1 (2)
	Mixed methods (quantitative and qualitative)	12 (27)
Outcomes measured/level		
	Reaction/satisfaction (Level 1)	25 (56)
	Knowledge (subjective) (Level 2A)	36 (80)
	Knowledge (objective) (Level 2B)	7 (16)
	Behavior/expertise (subjective) (Level 3A)	10 (22)
	Behavior/expertise (objective) (Level 3B)	2 (4)
	System results/performance (subjective) (Level 4A)	1 (2)
	System results/performance (objective) (Level 4B)	5 (11)

*Data missing for two articles (*n* = 43)

**Table 3:** Characteristics of 45 Curricula/Courses Addressing Leadership for Physicians in Studies Published Between 1989 and November 2013

Source (First author, year, reference number)	Setting	Learners	Intervention	Teaching methods	Educational content	Outcomes	Main findings
Awad, 2004^30^	Single U.S. residency program (surgery)	Surgery residents. Numbers not specified	Six months during residency	Not specified	A “focused program” to train residents to have the capacity/ability to create and manage powerful teams	Level 2A	Significant increase in score on a 34-item Internal Strength Scorecard: alignment (from 55 % to 68 %), communication (from 54 % to 66 %), and integrity (from 56 % to 68 %)
Babitch, 2006^31^	Single U.S. residency program (pediatrics)	Pediatrics residents (PGY1-3). Numbers not specified	Nine seminars	Seminars	A core curriculum focusing on physician compensation, economics, health care system, leadership and communication, career/CVs, contracts, health law, and customer service	Level 1; Level 2A	Satisfaction scores “between 3 and 4” on a four-point scale. Improvement in comprehension of the subject matter of each lecture (five-question scale), with an average increase of 20 % to 40 % between tests
Bayard, 2003^32^	Single U.S. residency program (family medicine)	Family medicine residents (PGY2-3). Numbers not specified	Two-year course. One half-day session per month	PGY2 residents work in groups on a simulated practice, interactive-lectures and assignments. PGY3 residents met for discussion	A practice management curriculum: Determining/balancing personal and professional goals, practice opportunities, facilities, organization, operation and management. Staff policies, legal issues, marketing, resources and hospital issues	Level 1; Level 2A	Self-assessed knowledge/comfort level of 13 topics on a five-point scale before and after the course. Average two-point increase in scores for all items
Bearman, 2012^33^	Single Australian residency program (surgery)	12 Australian surgery residents	Two-day course	Participants collected multi-source feedback from their workplace. Lectures, videos, simulation exercises, scenarios	Patient-centered communication, inter-professional communication, teamwork and professionalism	Level 1; Level 2A	Evaluation of the course using a five-point Likert scale instrument (*n* = 1), and free-text commentsreflecting self-perceived learning outcomes: leadership, teamwork, etc. Nine participants agreed or strongly agreed that they achieved each of the 14 learning objectives
Bergman, 2009^34^	Single Swedish hospital	53 managers (physicians, nurses, and other health personnel)	One week to 17 months	A one-week course and a long-term support group	Group dynamics, communication leadership	Level 2A; Level 3A	Questionnaire about coping abilities pre/post. Focus groups after the program. Both programs strengthened managers in their leadership roles. Increased self-awareness and improved communication
Bircher, 2013^35^	One UK deanery (extension of GP training)	GP trainees. Number not specified	Two-year program working on quality improvement and innovation projects, with support	Combination of experiential learning and taught program, tailored according to participants’ needs	The content was guided by the Medical Leadership Competency Framework, which includes domains of (1) delivering the service, (2) demonstrating personal qualities, (3) working with others, (4) managing services, (5) improving services, (6) setting direction	Level 2A	Method used is unclear. Qualitative material cited (“trainee feedback”) reflecting subjective learning outcomes
Block, 2007^36^	11 residency programs in Australia	146 participants (134 registrars and 12 resident medical officers)	Two-day program	Experiential small-group work, individual exercises, self-analysis questionnaires, videos, simulations, and some didactic content	Leadership competencies, self-awareness, communication and learning styles, conflict resolution, serving as teacher, time management, delegation, leadership styles, managing stress, safety and quality, team building, feedback and action planning	Level 1	High satisfaction with quality and content of presentations, with average score of 6.2 on a scale of 1 to 7
Boyle, 2004^37^	U.S., clinical leaders in two ICUs	Seven nurse and three physician leaders	Eight months, with six modules comprising a total of 23.5 hours	Training sessions for the leader group	Leadership, communication, coordination, problem solving/conflict management, and team culture	Level 2A; Level 2B; Level 3A; Level 4A	Communication skills of ICU nurse and physician leaders improved significantly. Leaders reported increased satisfaction with their own communication and leadership skills (Investigator-developed Collaboration Skills Simulation Vignette test and a modification of the ICU Nurse-Physician Questionnaire). Unit staff (six months after the interventions) reported increased problem-solving between groups and less perceived stress. Staff reported improved perceived quality of care
Brandon, 2013^38^	Single U.S. residency program (radiology)	44 radiology residents	One year	Seven modules, with lectures (90 min) and case-based group discussions	Costing analysis, fundamentals of improvement, practice groups and compensation, group practice selection, governance and management, process improvement, health care policy and economics, and negotiation and conflict management	Level 2A; Level 2B	Significant improvement in participants’ knowledge and self-assessed confidence scores for all modules (*p* < .001)
Cooper, 2011^39^	Single U.S. academic medical center	108 physicians, nurses, and allied health professionals	One-day training program	Two simulation and debriefing exercises in teams, seminar	Teamwork, patient safety, communication, individual and collective leadership	Level 1; Level 2A	High scores for relevance and quality of simulations on questionnaire and free-text comments. Statements during exercises were transcribed, and reflected subjective learning outcomes and insights about teamwork, communication, and leadership
Cordes, 1989^40^	Single U.S. residency program (preventive medicine and occupational medicine)	25 residents	One-month administrative rotation and project work	Practical experience from public health agencies (preventive medicine) and corporate settings and private practices (occupational medicine)	Budgeting, fiscal control, political processes and regulatory procedures, program development, personnel management, planning and organization, and computer skills	Level 2A; Level 4B	Participants’ overall rating of how beneficial the program was (score 3.23 on a four-point Likert scale). Participants’ careers were tracked, and 52 % had advanced to management positions
Crites, 2004^41^	Single U.S. residency program (internal medicine and pediatrics)	12 residents (PGY1-4)	Monthly seminar series covering 12 topics	Interactive lectures	Coding, regulatory issues, financial issues, human resource management	Level 2A; Level 2B	Participants scored higher on a self-assessed management skill, from 2.62 to 3.65 on a Likert scale of 1 to 5. Score on knowledge test increased from 74 % to 91 %
Dannels, 2008^42^	U.S./Canada executive education in academic medicine	78 women faculty at the associate or full professor level	One-year executive leadership development program for senior women faculty	Not specified	Executive leadership education	Level 2A; Level 3A; Level 4B	Change in pre/post intervention test of program participants (after 4–5 years) compared with two groups of women who did not participate in the program: a matched group from the AAMC faculty roster and a group of women who had applied to the program but had not been accepted. Program participants scored higher than comparison groups (*n* = 468) on 15 of 16 leadership indicators, including rank, position, leadership competencies and aspirations
Dougthy, 1991^43^	U.S., national program for pediatric chief residents	117 participants (over three years)	Three-day experiential workshop	Leadership training program with lectures and group exercises	Human interactions, stress management, management of teams and conflicts	Level 1; Level 2A; Level 3A	Participants rated satisfaction with the program components on a 10-point scale (mean score 8.2). 97 % said the conference would be useful to other chief residents. 20 of 67 attendees responded to six-month follow-up and reported changes in insight into personality types, ability to manage conflict, awareness of personal strengths/ weaknesses, ability to appreciate others’ perspectives, and ability to give negative feedback
Edler, 2010^44^	Single U.S. postgraduate pediatric anesthesiology fellowship	Not specified	One-year program during the first year of a residencyprogram	Experiential learning and training	Increased understanding of organizational culture and human factors, decision-making in technical planning, interpersonal or professional actions, and conflict resolution	Level 2A; Level 3B	Pre/post evaluation of residents’ leadership performance as scored by faculty on a Likert-type scale of 1–9, improved from 6.8 to 7.6 (*p* < .05). Qualitative evaluation (residents and faculty members) suggested improved clinical and administrative decision-making as learning outcomes
Evans, 1997^45^	Two U.S. residency programs (family medicine)	14 interns (PGY1) and 64 interns (PGY1) in a control group	One-day workshop and exercises in group development during intern rotation	Experiential, with outdoor activities and exercises	Group processes and teamwork skills	Level 2A	Respondents completed 27 questions designed to assess perceptions of trust, group awareness, group problem-solving, group effectiveness, and interpersonal communication. Study group scored higher on all main dimensions. Ten items, statistically significant higher score in intervention group (*p* < .05)
Gagliano, 2010^46^	Single U.S. hospital (physician organization)	90 physicians with some leadership responsibilities in their clinical practices	Two-year program with monthly sessions of 2–4 hours, three full-day intensive sessions (pilot), and a subsequent two-year program with four-hour monthly sessions	Lectures and case-based discussion	Organizational leadership, financial management, management strategy, applied skills and tools	Level 1; Level 2A; Level 3A	Each session was evaluated on a 5-point Likert scale. The majority of participants strongly agreed or agreed that the program as a whole had met expectations, was a valuable use of time and reported being better prepared for leadership responsibilities, and 79 % of participants reported that they had altered their approach to specific projects or problems because of the program
Gilfoyle, 2007^47^	Single Canadian residency program (pediatrics)	15 residents (PGY1–PGY4)	Half-day workshop	A plenary session followed by two simulated resuscitation scenarios	Tasks required of a leader, effective communication skills within a team, and avoidance of fixation errors	Level 2A; Level 3B	Learning was self-assessed using a retrospective pre/post questionnaire (five-point Likert scale) and revealed self-reported learning in knowledge of tasks, impact and components of communication, avoidance of fixation errors, and overall leadership performance (*p* < 0.001). Team performance was evaluated via a checklist. A second workshop was conducted after six months, and participants scored significantly higher compared with baseline and controls who had not participated in the first workshop
Green, 2002^48^	U.S. network of community-owned health care providers and physicians	26 teams from eight organizational units	Two-year coaching and leadership initiative	Four meetings, with team learning sessions and planning for six-month action period following the meetings. Teams from subsequent waves overlapped	Strategic goal-setting, engaging others, diffusion of innovation, PDSA, barrier-busting and infrastructure-building, project management, reflective thinking and learning, conceptual thinking, summarizing and communicating, coaching, and building further organizational capacity for spread	Level 4B	Participants scored the extent of the spread activities and sustainability of each project on a seven-point rating scale. Participating organizations tracked outcome metrics related to the goals of each improvement topic; 17 of 26 teams reported significant clinical improvements in targeted areas
Gruver, 2006^49^	Single U.S. health system	“Emerging leaders.” Numbers not specified	Duration not specified	Case-based leadership discussions during two-hour sessions	Managing vs. leading, forming a vision, predefining a person’s moral compass, risk-taking and transactional leadership	Level 1; Level 2A	Participants rated the program highly and reported learning outcomes (scores 3.88–4.78) on a five-point scale
Hanna, 2012^50^	Single Canadian residency program (surgery)	43 senior residents	One-day seminar	Case-based discussions, interactive lectures, real-life cases, live-feedback simulation role play, and revision of real contracts	Giving feedback and delegating duties, building teamwork, managing time, making rounds, coping with stress, effective learning while on duty, teaching at bedside and in the OR, and managing conflicts. Negotiating employment, managing personal finances, hedging malpractice risk, and managing a private practice	Level 2A	Evaluation with one questionnaire on how well topics were covered in their residency program, a second questionnaire on ability to perform nine managerial skills, and a third questionnaire assessing preparedness to perform managerial “duties” in future practice. For all managerial skills combined, 26 residents (60 %) rated their performance as “good” or “excellent” after the course vs. only 21 (49 %) before the course (*p* = .02)
Hemmer, 2007^51^	Single U.S. residency/fellowship program (pathology)	16 residents and fellows	One-year course	Six sessions (average 10 hours per session). Didactic lectures, interactive sessions, case scenarios, team-building exercises, formal team presentations (capstone project)	Fundamental principles of laboratory administration, managing change and interpersonal skills, personnel issues and quality, informatics, and finance	Level 1; Level 2B	Participants evaluated (five-point scale) the content and speakers (scores from 4.4 to 5.0). Pre/post course assessment in which participants showed significant improvement in their leadership and management test scores (from 61 % to 88 % (*p* < .002) and from 61 % to 88 % (*p* < .001) in two different cohorts)
Kasuya, 2001^52^	Single U.S. residency program (internal medicine)	Residents (PGY1). Number not specified	One-day retreat	Six-hour program. Lectures and small-group tasks and discussions, scenarios and role play	Setting personal vision, leadership vs. management, building a team, practical negotiation skills, providing effective feedback, and problem-solving as a team leader	Level 2A	Participants completed entry and exit questionnaires responding to items using a four-point Likert scale (4 = strongly agree to 1 = strongly disagree). Increased confidence in their abilities to lead a ward team (*p* = .0002) and fulfill their responsibilities as upper-level residents (*p* = .0002), and having identified qualities they aspired to as upper-level residents (*p* = .0014)
Kochar, 2003^53^	Single U.S. academic medical center	30 faculty members	Nine-day course in three-day segments over five months	Sessions, lectures	Managing people, health care finance and accounting, leadership, marketing, health care informatics and information technology, health care quality, health care economics, time management	Level 1	Participants rated the sessions in 12 dimensions on a scale of 1 to 5, with average scores of 4.2 to 4.6
Korschun, 2007^54^	Single U.S. academic medical center	70 participants, including 29 physicians	Five three-day sessions over five months	Lectures, case studies, experiential exercises, individual assessment, executive coaching, including a 360° assessment. Project team of 5–6 members worked on a project. Each fellow paired with a mentor	Strategic thinking and personal awareness, leadership qualities and best practices, negotiation and conflict management, collaboration, marketing, change management, and crisis management	Level 1; Level 2A; Level 3A; Level 4B	Evaluation of each session, surveys after each year’s program and an online survey after year 3 of program. Participants reported positive experiences with the program and reported skills and competencies. Mentoring received lower scores than other components. 57 % had modified career goals, 15 % had been promoted, 56 % had been given additional responsibilities, and 76 % reported taking on additional leadership responsibilities. Group projects were assessed and organizational outcomes were identified (such as increased patient satisfaction)
Kuo, 2010^55^	Single U.S. residency program (pediatrics)	24 residents (PGY1-PGY3)	Three-year longitudinal program incorporated in residency training	Small-group seminars, project work, and mentoring	Personal leadership development, team-building, negotiation, and conflict management	Level 1; Level 2A	Entrance survey and exit evaluation. Scores on a scale of 1 to 4: satisfaction with program (3.73, impact on long-term career goals (3.55), positive impact on plans to influence population health and health policy (3.53), positive impact on plans to serve minority or underserved (3.47), improvement of competence as a leader (3.40). Supplemented with process evaluation and feedback from faculty and participants
Levine, 2008^56^	Single U.S. academic medical center (chief residents in medicine and surgery)	47 chief residents	Two-day offsite immersion training	Small-group case discussions, mini-lectures, seminars, one-on-one mentoring to develop a project	Foster collaboration between disciplines in the management of complex older patients, increase knowledge of geriatric principles, enhance eadership skills (giving feedback, approaching the reluctant learner, conflict resolution)	Level 2A; Level 2B	Evaluation included pre/post program tests and self-report surveys and two follow-up surveys or interviews. Mean enhancement was 4.3 (on a scale of 1 to 5)
LoPresti, 2009^57^	Four U.S. residency programs (family medicine)	20 residents (PGY2)	12-month simulated practice (*n* = 6) training vs. standard program (*n* = 14)	Lectures, in-class exercises, group work in 20 modules (60 hours)	Leadership, negotiation, and an array of practice management competencies	Level 2A; Level 2B	Pre-test and post-test examinations with a control group. Residents in the intervention group had statistically significant increases in exam scores, while the comparison group did not. The simulated practice group also increased scores on every subsection of the exam, while the comparison group increased scores on only half of the subsections. Competency in leadership did not improve, with pre/post scores of 39 % and 40 %, respectively, in the intervention group and 43 % and 39 %, respectively, in the control group
McAlearney, 2005^58^	Single U.S. hospital	52 physicians (two cohorts)	Two-year longitudinal program	Format: 20 months. Hourly sessions/interactive seminars monthly, and half-day sessions every half-year	Leadership, teamwork, transformational change, strategic planning, conflict resolution, delegation, finance, business of health care	Level 1; Level 2A; Level 3A	Survey among participants in first cohort one year after the two-year program (on a scale of 1–5, strongly disagree–agree): more effective in their leadership roles (4.2), more effective working in teams (4.0), more effective leading teams (4.3), and experienced opportunities to expand leadership roles after program (4.0). Qualitative evaluation indicated impact on leadership behaviors
Murdock, 2011^59^	Program involving three U.S. states	100 community practice physicians (five cohorts)	20-week program	Weekly three-hour evening sessions	The business of medicine, quality improvement, and transformational leadership	Level 2A	Survey at entry and exit. Physicians self-assessed their levels of skills and competencies. Increase in self-assessed competency in all of the 26 categories in each of the program’s five cohorts
Mygdal, 1991^60^	Program involving one U.S. state (family medicine)	27 residents (PGY2) who would be serving as chief residents in PGY3	Conference. Duration not specified	Two workshops, group discussions, plenary speeches, and a concluding planning session	Leadership and stress-coping skills, and exposure to organized medicine	Level 1; Level 2A	Self-rating five-point Likert scale. Participants completed a five-item reactions to conference scale and 10-item self-rating scale (pre/post event). Residents reported favorable reactions to conference (4.33) and reported a perception that it helped their abilities in stress management and leadership. There was an increase of 1.29 points in self-evaluation of skills
O’Donnel, 2011^61^	Single U.S. hospital (residency programs)	Residents (PGY1). Numbers not specified	Four-week rotation/program in a department of case management	Two-hour class over four weeks	Promote physician knowledge and awareness of financial and quality implications of health care delivery as a comprehensive team	Level 1	Feedback on program content (92–100 % of objectives met)
Patterson, 2013^62^	Four UK training programs for general practice	Third-year GP residents. Numbers not specified	Eight-month cross-regional program	Practice-based project, information-sharing meeting, and five facilitating meetings	Leadership, change management, and teamwork skills	Level 1; Level 2A	MLCF questionnaires before and after the program. Higher scores on self-awareness, but no data provided. Quotes from qualitative material reflect a wish for more structure and formal training
Pearson, 1994^63^	Single U.S. residency program	Junior and senior residents. Numbers not specified	Two six-week blocks during primary care rotation	12 sessions, one per week, during the residents’ two primary care blocks	Resident as manager, leadership, interpersonal skills, delegating; continuous quality improvement, coaching and organizational culture	Level 1; Level 2A	A continuous process that included Likert-scale and written evaluations at the end of each year, and a final oral self-assessment by each resident. Overall satisfaction with the program was 6 on a scale of 1 to 7. Oral and written evaluation indicated “great value” of the program
Pugno, 2002^64^	U.S. residency director program	Residency directors (family practice). Numbers not specified	Nine-month program	A three-day conference and two one-day sessions. Project work during nine-month period with mentor-advisor	Leadership development, resource allocation, familiarity with regulations and standards, educational options, and personnel management skills	Level 1; Level 2A	Survey among previous participants (41 % of 241); 85 % rated it “very valuable,” 14 % rated it “valuable,” 76 % reported that the program lowered the level of stress, 22 % reported that it had no impact, and 2 % reported that it raised the level of stress
Richman, 2001^65^	U.S./Canada executive education in academic medicine	Several cohorts of women faculty at the associate or full professor level	One-year experiential executive leadership development program	Three sessions, interactive teaching methods, lectures, panel discussions, case studies, computer simulations, role play, small-group work, individual projects, and 360° feedback	“Mini-MBA” (fiscal planning and budgeting, resource management and allocation, etc.), emerging issues, and personal development (conflict management and negotiation skills, team-building skills development through small-group projects)	Level 2A; Level 2B; Level 4B	Mixed methods. Pre-program and post-program data (knowledge tests). Program evaluation (qualitative) and career tracking. Participants have been successful in advancing to higher leadership roles. Pre/post program test (*n* = 77) found significant improved score for all curricular areas: financial management, career advancement, personal leadership, converging paradigms of academic and corporate leadership, emerging issues, and strategic planning (*p* < .0001)
Singer, 2011^66^	Single U.S. academic medical center	12 multidisciplinary management groups (*n* = 108)	15 months	Four sessions (team-based learning, simulation, and project management exercises) and a final interview	Team-based leadership behaviors	Level 3A	Transcripts from sessions suggested that the training prompted personal insights, greater awareness, and exercise of leadership behaviors among participants. Average of 8.4 on a scale of 0–10 impact on targeted leadership behaviors
Steinert, 2003^67^	Single Canadian department	16 medical faculty (family medicine)	Two-day workshop	Interactive modules and exercises	Time management, determining goals and priorities, leadership styles and skills, and conducting effective meetings	Level 1; Level 2A; Level 3A	Post-workshop questionnaire administered to participants. Workshop rated as “very useful” by all. One year later, 10 participants were interviewed to explore behavioral changes. Self-assessed positive change for determining short-term goals, handling paper more effectively, protecting time, and setting meeting agendas. They were less successful at delegating, saying no, adopting different leadership styles, and evaluating meetings
Stergiopolous, 2009^68^	Single Canadian residency program	Junior residents (PGY-2) (*n* = 24) and senior residents (PGY-4) (*n* = 28)	Workshops (four half-days)	Didactic teaching and small groups (buzz groups, think-pair-share discussions, a debate, and clinical case studies)	Evaluation, leadership and change management, mental health reform, teamwork, conflict resolution, quality improvement, program planning	Level 1	At the end of each workshop. residents completed an anonymous form querying about the importance and clinical usefulness of the objectives, rated on a five-point Likert scale, as well as open-ended comments about the strengths and weaknesses of the workshops and suggestions for improvement. High satisfaction scores (4.19–4.33)
Stoller, 2004^69^	Single U.S. residency program (internal medicine)	Junior residents (PGY-1) (*n* = 32)	One-day retreat	Team-building exercise, group discussion	Team skills, group dynamics, leadership	Level 2A	Baseline and follow-up questionnaires suggest that the retreat enhanced participants’ self-assessed ability to be better physicians, resident supervisors, and leaders
Stoller, 2009^70^	Single U.S. hospital	Physicians	Nine-month program with 9–10 days/year	Lectures, project work in groups, development of business plans	Accounting, financing, marketing, leadership, human resource management, emotional intelligence, negotiation, conflict resolution	Level 4B	Review of business plans developed shows that a total of 49 business plans were submitted, and 30 (61 %) have either been implemented or have directly affected program implementation at the clinic
Vimr, 2013^71^	Single Canadian hospital	Physician leaders. Numbers not specified.	Eight months, five 1.5-day meetings	Lectures, self-reflection, action learning projects (in teams) and coaching	Alignment of competencies, a systems and collaborative approach, affective learning strategies	Level 1; Level 2A	Quantitatively, the average rating for all components was 4.64 on a 6.0-point Likert scale. Qualitatively, participants reported on how they had changed as a person, and what they would do differently
Weiss, 1992^72^	Single U.S. residency program	Three residents (pathology)	One-month elective	Four hours of lectures, and the rest group exercises	Finance and accounting, general, human resource, and operations management	Level 1; Level 2B	Evaluation of course content (well-received). Pretest-post-test MCQ (25 items). Scores increased from 67 % to 83 %
Wisborg, 2006^73^	28 Norwegian hospitals	Multi-professional training course for hospital trauma teams (*n* = 2,860)	One-day training session	3.5-hour didactic session with theory and discussions, followed by practical training in the hospitals’ trauma room	Communication, cooperation, and leadership	Level 1; Level 2A	Pre/post-course: self-rated knowledge outcome on a 1–10 VAS scale. Respondents who participated in the simulation and debriefing scored the learning and fulfilment of expectations higher than those who took part in the didactic session only. Of the 1,237 that participated in the practical simulation, 99 % found the session to be a valuable learning experience
Wurster, 2007^74^	Single U.S. hospital	42 fellows (surgeons, nurses, and directors)	Six-month program	Weekend of didactic study, followed by six months of teamwork on projects, monthly conferences, and two days for presentations	Patient safety, leadership and management skills	Level 1; Level 2A	Survey pre/post. Baseline surveys on leadership skills knowledge, patient safety knowledge, and program goals. Completed the same surveys seven months later. Results for patient safety post-program were significantly higher for 8 of 10 questions. All results were significantly higher for leadership

A total of 29 articles described programs for physicians (including residents and faculty) without a formal leadership role, and 16 articles described programs for individuals in formal leadership roles (chief residents, physicians with leadership responsibilities, program directors, and faculty in leadership position) (Table 3). The duration of training ranged from a half-day workshop^47^ to a three-year program.^55^ Most programs (*n* = 32) were delivered as an extended course, most often over a period of 12 months; fewer (*n* = 13) were one-time events (such as a single workshop, conference, or a course).

### Educational Aims and Content

The educational programs in more than half of the 45 studies focused on training skills, including exercises on giving feedback, building teams, resolving conflicts, communicating and writing a business plan, or teaching technical and conceptual knowledge (Table 2). Personal growth and self-awareness were explicit aims in nine programs. The curricula addressed a wide range of educational content and displayed great diversity—and, at times, inconsistency—in concepts of leadership and management (see Tables 2 and 3). The most common topics included in the curricula were leadership, teamwork, financial management, self-management, conflict management, quality improvement, communication, and health policy/strategy.

### Teaching/Learning Methods

Teaching methods were specified in 43 articles (Table 2), while two articles lacked this information.^30^^,^ 42 Of the 43 programs, 36 used didactic lectures/interactive plenary seminars, 32 involved group work (case-based discussions, exercises, group reflections), 16 included project work (action-based learning, project planning), and 12 reported the use of simulation exercises (simulated practice and role play). Multi-source feedback or a 360-degree feedback tool was used in three programs.^33^^,^ 54 ^,^ 64 Most of the programs used two or more teaching/learning methods in the curricula.

### Evaluation Design and Outcomes

About half of the 45 studies used pre/post intervention designs as the basis for evaluating outcomes. Most post-intervention assessments occurred immediately after the program, while five studies assessed participants over a longer time span, of which three programs scored participants at baseline and at six months post-intervention,^34^^,^ 37 ^,^ 47 and three reported data on participant career development.^40^^,^ 42 ^,^ 64 Only five studies^34^^,^ 42 ^,^ 45 ^,^ 57 used a comparison group. Quantitative data only (surveys, tests, standardized observations, etc.) were used in the majority of studies, and qualitative data only (free-text comments, oral evaluation, and semi-structured interviews) were used in one study.^35^ Mixed evaluation methods were used in one-fifth of the studies.

A majority (*n* = 25) of the articles reported participants’ reaction scores (Level 1), and four-point or five-point Likert scales were commonly used to rate modules, sessions, or the program as a whole (Tables 2 and 3). Self-assessed knowledge outcomes (Level 2A) were reported in 36 of the studies, while objective tests of knowledge (Level 2B) were used alone or in addition to self-assessed measures in seven of the studies. Self-assessed learning outcomes for behavior/expertise (Level 3A) were reported in 10 studies. Two studies reported using objective outcome measures for behavior/expertise (Level 3B), such as using a form to score a third person’s leadership performance^44^ or using a checklist to score a team’s performance.^47^

Outcomes at the system (e.g., organizational) level (Level 4A and Level 4B) were reported in six articles. Staff-assessed increased quality of care was measured in one of these studies,^37^ and participant success in advancing to higher leadership roles was reported in three studies.^42^^,^ 54 ^,^ 64 Two of the studies documented objective outcomes on quality indicators for management of diseases such as diabetes, asthma, and breast cancer,^48^ and one study reported increased customer satisfaction.^54^ One study measured the number of business plans implemented.^70^

## DISCUSSION

We identified 45 peer-reviewed articles that described and reported evaluation outcomes of physician leadership development programs. We found considerable heterogeneity concerning conceptual frameworks, teaching and learning methods, educational content, evaluation design, and outcomes measured. Most programs identified in this study targeted either resident physicians with no formal leadership roles or physicians in mid-level management positions. We found no reports on programs for physicians in top-level leadership positions. Almost two-thirds of the programs focused on skills training and technical and conceptual knowledge, while one-fifth of the programs focused on personal growth and awareness. All 45 studies reported positive outcomes, but few studies reported system-level effects, such as improved performance on quality indicators for disease management or increased customer satisfaction.

As a whole, the reports in the literature indicated that the majority of programs targeted physicians exclusively, with no participation of other professional groups within the health care organization. Although experts have noted that physician-only programs may facilitate open dialogue among participants^75^, it is possible that such approaches miss opportunities for developing the capacity to collaborate across professional lines, which may be important for team-based leadership.^26^^,^ 27 This review suggests that current approaches to physician leadership development focus more on the skills of individual physicians than on enhancing the capacity for collaboration through cultivating greater levels of understanding and communication networks across professional groups.

We also found that although self-awareness within larger groups and organizations is fundamental to leadership capacity, relatively few programs addressed personal growth and self-awareness.^19^^,^ 20 ^,^ 22 ^,^ 25 One-third of the programs addressed self-management, but the methods were limited, and few programs reported using any sort of multi-source feedback tool. Our findings suggest that the leadership programs described in the medical literature focus more on the “know” and “do” elements of leadership than the “be” component, which some argue is fundamental in attaining the capacity to lead.^19^^,^ 20 ^,^ 25 As teamwork and collaboration are increasingly required in the area of health care, there is a growing need to include self-awareness and emotional intelligence as fundamental competencies within leadership development programs.^9^^,^ 10 ^,^ 13 ^,^ 76

We found that programs largely employed lectures, seminars, and group work rather than the broader set of teaching tools available for leadership development, including developmental relationships (mentors, coaching, peer learning partners), assignments (job moves and rotations, action-based learning projects), feedback processes (performance appraisal, 360° feedback), and self-developmental activities.^15^^,^ 20 ^–^ 22 ^,^ 75 This finding is consistent with the recognition that, thus far, the literature on physician leadership development has been centered on imparting conceptual knowledge to physicians as individuals, for which lectures and seminars may be suitable, and has directed fewer resources to efforts in building self-awareness, for which action-based learning, feedback, and self-development activities may be more appropriate. Importantly, the few studies that documented favorable organizational outcomes, such as improvement in quality indicators for disease management, were characterized by the use of multiple learning methods, including lectures, seminars, and group work, and involved action learning projects in multidisciplinary teams.^42^^,^ 48 ^,^ 54 The implication of this finding is that greater investment in programs using teamwork and multiple learning methods is likely to have the largest impact in the area of leadership development for physicians. And while these may be more expensive and time-consuming to undertake, real progress will likely require such resources, and lower-level efforts may continue to have a limited effect.

Furthermore, we found that most of the literature evaluated the impact of programs on a narrow set of measures, most commonly participant satisfaction scores and self-assessed knowledge and behavioral change. Only six studies examined more complex outcomes at the system level. Evidence from outside the medical field has indicated that leadership development activities can positively influence organizational performance;^24^^,^ 25 however, the evidence base remains modest due to the paucity of studies that have assessed organization-level outcomes. Pilot programs are needed, with robust evaluation, to provide a base of evidence for the most effective means of achieving this critical capacity. We have come a long way in calling for great leadership among physicians, but there is opportunity for further improvement. Although learner satisfaction and individual learning outcomes are important, there is a dearth of research exploring clinical outcomes and organizational effects, as well as a lack of studies exploring the mechanisms by which leadership programs foster learning and change.

Our findings should be interpreted in light of several limitations. First, many of the studies we reviewed exhibited weak study design, modest and selected samples of participants, and a limited scope of outcomes measured. Furthermore, there was substantial heterogeneity among evaluation designs, outcome measures, and conceptual frameworks, precluding a quantitative synthesis of the varied findings. Although these are acknowledged limitations, this recognition also provides an understanding of the current state of evidence and highlights important paths for improvement with regard to studies on physician leadership development. Second, we limited our search to the peer-reviewed literature, excluding data on programs reported in the grey literature. Although this may have resulted in our missing novel programs, we wanted to ensure an adequate understanding of the methodologies employed, and thus focused on peer-reviewed scientific literature. Last, our findings likely suffer from publication bias, in that negative studies that have shown no significant impact of leadership development programs were likely underrepresented in our review. This is a common challenge for reviews of peer-reviewed literature, and is important to acknowledge in interpreting our findings.

In conclusion, the literature indicates that physician leadership development programs are associated with significantly increased self-assessed knowledge and expertise among physician participants; however, few studies have examined the impact on broader outcomes at an organizational or system level. Furthermore, our synthesis of the literature suggests important gaps, including a lack of programs that integrate non-physician and physician professionals, a limited use of more advanced training tools such as interactive learning and feedback in order to develop greater self-awareness, and an overly narrow focus on individual-level rather than system-level outcomes.
